# Epigenetic Regulation of Cardiac Troponin Genes in Pediatric Patients with Heart Failure Supported by Ventricular Assist Device

**DOI:** 10.3390/biomedicines9101409

**Published:** 2021-10-07

**Authors:** Rosetta Ragusa, Arianna Di Molfetta, Serena Del Turco, Manuela Cabiati, Silvia Del Ry, Giuseppina Basta, Alberto Mercatanti, Letizia Pitto, Antonio Amodeo, Maria Giovanna Trivella, Milena Rizzo, Chiara Caselli

**Affiliations:** 1 Institute of Clinical Physiology, CNR, 56124 Pisa, Italy; rragusa@ifc.cnr.it (R.R.); serena@ifc.cnr.it (S.D.T.); manuela.cabiati@ifc.cnr.it (M.C.); delry@ifc.cnr.it (S.D.R.); lapina@ifc.cnr.it (G.B.); alberto.mercatanti@ifc.cnr.it (A.M.); l.pitto@ifc.cnr.it (L.P.); trivella@ifc.cnr.it (M.G.T.); milena.rizzo@ifc.cnr.it (M.R.); 2 Scuola Superiore Sant’Anna, 56127 Pisa, Italy; 3 Departement of Cardiothoracic Surgery, Ospedale Pediatrico Bambino Gesù, 00165 Rome, Italy; arianna.dimolfetta@gmail.com (A.D.M.); antonio.amodeo@opbg.net (A.A.); 4 Fondazione Toscana Gabriele Monasterio, 56124 Pisa, Italy

**Keywords:** Heart Failure, VAD, NGS, cardiac miRNA, cardiac troponin, pediatric patients

## Abstract

Ventricular Assist Device (VAD) therapy is considered as a part of standard care for end-stage Heart Failure (HF) children unresponsive to medical management, but the potential role of miRNAs in response to VAD therapy on molecular pathways underlying LV remodeling and cardiac function in HF is unknown. The aims of this study were to evaluate the effects of VAD on miRNA expression profile in cardiac tissue obtained from HF children, to determine the putative miRNA targets by an in-silico analysis as well as to verify the changes of predicated miRNA target in the same cardiac samples. The regulatory role of selected miRNAs on predicted targets was evaluated by a dedicated in vitro study. miRNA profile was determined in cardiac samples obtained from 13 HF children [median: 29 months; 19 LVEF%; 9 Kg] by NGS before VAD implant (pre-VAD) and at the moment of heart transplant (Post-VAD). Only hsa-miR-199b-5p, hsa-miR-19a-3p, hsa-miR-1246 were differentially expressed at post-VAD when compared to pre-VAD, and validated by real-time PCR. Putative targets of the selected miRNAs were involved in regulation of sarcomere genes, such as cardiac troponin (cTns) complex. The expression levels of fetal ad adult isoforms of cTns resulted significantly higher after VAD in cardiac tissue of HF pediatric patients when compared with HF adults. An in vitro study confirmed a down-regulatory effect of hsa-miR-19a-3p on cTnC expression. The effect of VAD on sarcomere organization through cTn isoform expression may be epigenetically regulated, suggesting for miRNAs a potential role as therapeutic targets to improve heart function in HF pediatric patients.

## 1. Introduction 

Pediatric Heart Failure (HF) is a complex condition characterized by genetic, structural and neurohormonal abnormalities, resulting in ventricular dysfunction, volume or pressure overload, impaired oxygenation of organs and tissues [1,2]. The reported incidence of pediatric HF is 0.97 to 7.4 per 100,000 and it was estimated that more than 11,000 children are hospitalized for HF each year [3]. Unlike HF in adult patients, congenital heart disease (CHD) and dilated cardiomyopathy (DCM) have been identified among principal etiologic categories that favor the development of HF and the principal reasons for heart transplant in pediatric patients [4]. A dedicated trial aimed to verify the best medical treatments for HF children has not been performed yet, and drugs used in pediatric patients are mainly derived from adult studies, although the underlying etiologies of HF are very different. Accordingly, beta-blockers and anticoagulation medication were included as medical treatment also in pediatric patients in the attempt to reduce the HF progress, minimizing morbidity and mortality and improving the quality of life [5,6]. The application of ventricular assist device (VAD) therapy, as bridge to heart transplant, is now considered as a part of standard care also for end-stage HF children unresponsive to medical management [5,6]. 

In recent years, molecular mechanisms underlying HF in children have been studied and cardiac fetal genes re-expression (genes of sarcomere components) or epigenetic modifications (DNA methylation, ATP-dependent chromatin remodeling, histone modifications, and microRNA-related mechanisms) have emerged as factors inducing left ventricle (LV) remodeling and HF progression [5,7]. Among them, microRNA (miRNA), small non-coding RNAs (~22 nucleotides) with a role in regulating gene/protein expression [5,7], are expressed during normal growth from embryonic, postnatal to adult hearts and their aberrant expression or genetic deletion is associated with abnormal cardiac cell differentiation, cardiac dysfunction [8], hypertrophy and fibrosis [9,10]. In human, the cardiac expression levels of some miRNAs were impaired in end-stage HF adult patients compared to healthy subjects [11], and cardiac miRNA profile of adult HF patients supported by VAD, as bridge to transplant, was different from that of HF patients directly submitted to transplantation, suggesting a potential effect of mechanical heart unloading on molecular mechanisms underlying ventricular remodeling [12]. Investigation on the potential role of miRNAs in heart remodeling, disease progression and response to therapy in HF children are limited and require more attention [13,14].

Thus, the aim of this study was to evaluate changes in cardiac miRNA profile in heart samples collected from pediatric patients supported by VAD. Specifically, the cardiac miRNA profile of HF children was determined by NGS at VAD implant and then at heart transplant. Moreover, putative targets of differentially expressed miRNAs were identified by an in-silico analysis and their changes were evaluated in cardiac samples. Finally, the regulatory role of selected miRNAs on predicted targets was evaluated by a dedicated in vitro study.

## 2. Materials and Methods

### 2.1. Experimental Design

**Figure 1:**
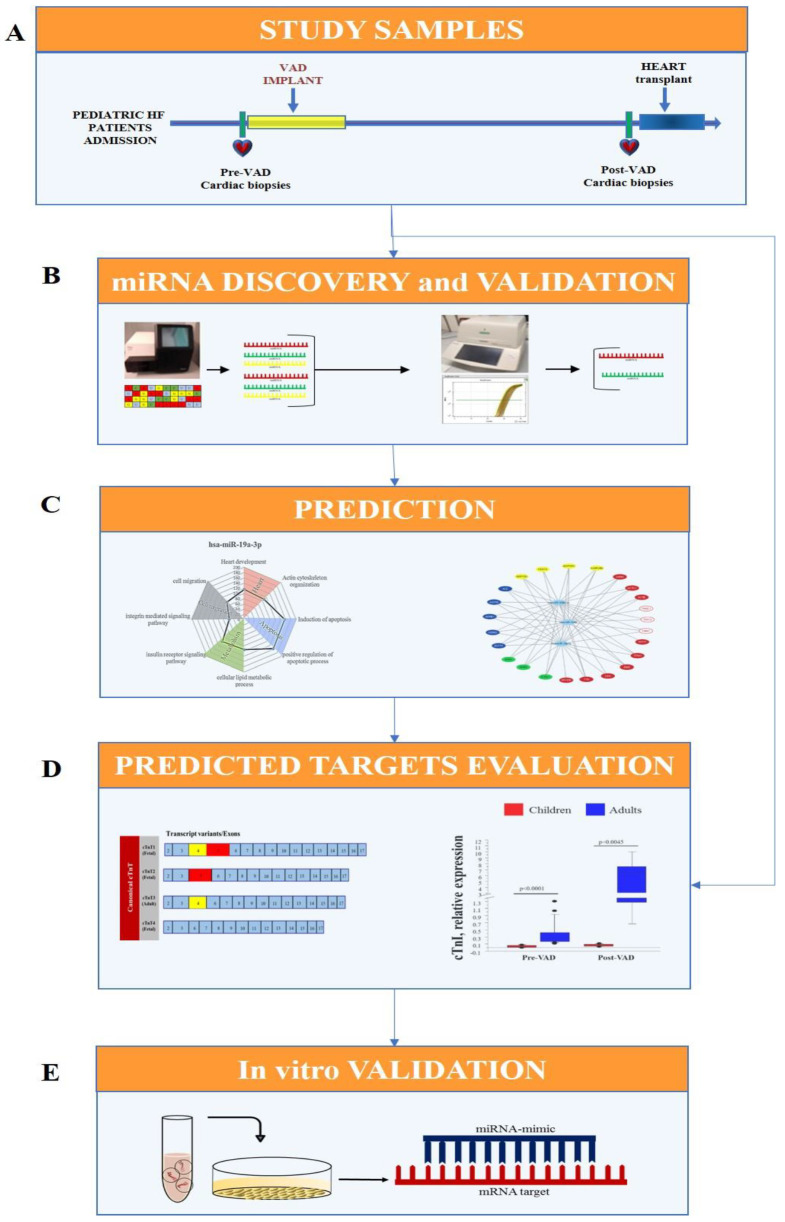
Experimental design. (**A**) Cardiac samples were collected from HF pediatric patients at the moment of VAD implant (pre-VAD) and at the moment of heart transplant after VAD support (post-VAD). Total RNA (miRNA and mRNA) was extracted from cardiac samples. (**B**) In order to identify the miRNAs modified by VAD support, cardiac miRNA profile at pre-VAD and post-VAD was evaluated by NGS and real-time PCR was performed to validate sequencing results. (**C**) The in-silico analysis was employed to predict the putative targets of validated miRNAs. (**D**) The cardiac expression of miRNAs predicted targets was evaluated in HF children at pre-VAD and post-VAD and compared with a group of adults with HF. (**E**) An in vitro transfection study in HL-1 cell line was performed to verify the regulatory role of selected miRNAs on predicted targets.

The experimental design of this work included the following steps (Figure 1):

- Cardiac samples were collected from pediatric HF patients at VAD implant (pre-VAD) and at heart transplant (post-VAD);
- Cardiac miRNA profile was performed by Next Generation Sequencing (NGS) in pre-VAD and post-VAD samples and sequencing results were confirmed by real-time PCR and the validated miRNA were selected for future analysis;
- Sarcomere components, including cardiac troponins, were identified as putative targets of selected miRNAs by an in-silico analysis;
- Variation in expression of cardiac troponins were evaluated by real-time PCR in cardiac biopsies from HF pediatric and adult patients, as previous reported [12,15,16];
- An in vitro transfection study using miRNA mimic in HL-1 cell line was carried out for testing the regulatory role of selected miRNA on predicted targets.

### 2.2. Study Sample

The study population included 13 HF pediatric patients undergoing VAD implantation as bridge-to transplantation at the Cardiovascular Department of Ospedale Bambino Gesù of Rome. *N* = 9 patients were implanted with pulsatile-flow pump (Thoratec, Berlin Heart Excor), *n* = 3 patients were implanted with continuous-flow pump with Jarvik, *n* = 1 patient was supported with biventricular assist device (BiVAD; Thoratec, Berlin Heart Excor). Clinical and echocardiographic data were collected at VAD implant and at 1 month after implantation. This study complied with the principles of the Declaration of Helsinki. Informed consent was given by all parents of children enrolled in this study and the protocol was approved by the Ospedale Bambino Gesù Ethic Committee.

Cardiac biopsies were collected at the time of VAD implantation (pre-VAD) from the portion of left ventricle (LV) apex excised during standard surgical procedure (needed for inflow cannula positioning) and at the time of heart transplant (post-VAD) from LV and septum. Sample collection included *n* = 8 myocardial biopsies at pre-VAD and *n* = 7 at post-VAD (*n* = 5 biopsies from LV and *n* = 2 biopsies from septum). Immediately after collection, myocardial samples were frozen in liquid nitrogen and stored at −80 °C until sample preparation.

In order to compare expression levels of sarcomere genes from HF pediatric patients with those obtained from HF adult patients, data derived from cardiac samples of patients previously recruited at the CardioThoracic and Vascular Department of Niguarda Ca’ Granda Hospital in Milan (Italy) were utilized [12,15,16]. The adult population included a group of end stage adult HF supported with VAD as bridge-to transplantation [60 (50–64) years, 14 males, 22.5 (19.5–25) left ventricular ejection fraction (LVEF)%]. Cardiac samples were obtained from LV apex (*n* = 16) to pre-VAD and from LV (*n* = 5) to post-VAD. Immediately after collection, myocardial samples were frozen in liquid nitrogen and stored at −80 °C until sample preparation.

### 2.3. RNA Extraction

Total RNA was extracted from heart samples using acid guanidinium-thiocyanate-phenol-chloroform method (*miRNeasy Mini Kit*, Qiagen S.p.a, Milano, Italy). RNA concentration and purity were determined spectrophotometrically (NanoDrop, Thermo Fisher Scientific, Milano, Italy). RNA integrity was evaluated by 2100 Bioanalyzer (Agilent Technologies, Milano, Italy). The total RNA was stored to −80 °C until use.

### 2.4. Libraries and NGS

The small cDNA libraries construction was performed with *TruSeq small RNA sample preparation Kit* (Illumina, San Diego, CA, USA) from 1µg of total RNA extracted from cardiac biopsies obtained at pre-VAD and at post- VAD implant. The cDNA libraries were loaded at eight-plex level of multiplexing into a flow cell V3 and sequenced in a single-reads mode (50 bp) on a MiSeq sequencer (Illumina, San Diego, CA, USA). Raw data were analyzed and miRNA identification were performed as previous described [17].

### 2.5. Bioinformatic Analysis of Identified miRNAs

miRNAs count matrices were analyzed using R Bioconductor’s package DESeq2 [18]. miRNAs with Benjamini and Hochberg (1995) adj-*p* value < 0.05 were selected for downstream validation step using real-time PCR. The principal component analysis (PCA) was performed with R package BiocGenerics v 0.30.0 (R Foundation for Statistical Computing, Institute for Statistics and Mathematics, Vienna, Austria) using the normalized read counts to verified the distribution of miRNA among pre-VAD and post-VAD groups. 

### 2.6. NGS Data Validation 

To confirm data obtaining by NGS, a validation study by real-time PCR was performed. The reverse transcription reaction of 1 μg miRNA from total RNA has been performed using *miScript II RT Kit* (Qiagen S.p.a, Milano, Italy). Real-time PCR were performed in duplicate in the Bio-Rad C1000 thermal cycler (CFX-96 Real-Time PCR detection systems; Bio-Rad Laboratories, Hercules, CA, USA) using *SsoFAST EvaGreen Supermix* (Bio-Rad Laboratories, Hercules, CA, USA). Amplification of cDNAwas carried out using primer pairs formed by the miScript Universal primer (Qiagen S.p.a, Milano, Italy) and a primer having the same miRNA sequence to be analyzed (Table S1 of the Supplementary Material). Normalization of miRNAs levels was performed using the endogenous reference gene U6.

### 2.7. Bioinformatic Prediction of miRNA Targets

A previous reported [19], predicted miRNAs-gene targets were selected using miRWalk 2.0 database (http://zmf.umm.uni-heidelberg.de/apps/zmf/mirwalk2, 5 May 2021), which provides the largest available collection of miRNA-target interactions obtained from 12 established prediction algorithms. For each list of putative targets, the Gene Ontology (GO) analysis was performed to identify the enriched biological processes (BP) in which miRNAs could be involved. An adjusted (adj)-*p* value < 0.05 was considered statistically significant.

### 2.8. Troponin Expression in Cardiac Tissue

To design primers for the transcript variants of cardiac troponin T (canonical and non-canonical), an in-depth study by Universal Protein knowledgebase (UniProt) and Ensembl (EMBL-EBI-European bioinformatics Institute) was carried out. The UniProt, a database of protein sequences (http://www.uniprot.org, 5 May 2021), was employed to evaluate the number of cardiac troponin T (cTnT) isoforms and the length of each sequence. This information was used to identify the correspondent transcript variants using Ensembl (https://www.ensembl.org/index.html, 5 May 2021, European Molecular Biology Laboratory's European Bioinformatics Institute, Wellcome Genome Campus in Hinxton, Cambridge, United Kingdom). The reverse transcription of mRNA to cDNA was performed using *IScript cDNA Synthesis Kit* (Bio-Rad Laboratories, Hercules, CA, USA) optimized for reliable cDNA synthesis over 1 μg of mRNA as template. The expression of cardiac troponins (cTns) was analyzed by CFX-96 Real-Time PCR detection systems (Bio-Rad), starting from 2 μL cDNA template and *SsoFAST™ EvaGreen^®^ supermix* (Bio-Rad Laboratories, Hercules, CA, USA). Primer-Blast (National Center for Biotechnology Information) was used for designing reference and gene target primers (Table S2). Considering the high similarity of the non-canonical troponin T sequences, it was not possible to identify a specific primer pair for each non-canonical cTnT. For this reason, a primer pair able to amplify a region present in both cTnT10/cTnT11/cTnT12 was used. Moreover, a specific primer pair for cTnT12 was designed with Primer-Blast and inserted into the Table S2. Normalization of cTns mRNA was carried out using tryptophan 5-monooxygenase activation protein zeta polypeptide (YWHAZ), ribosomal protein L13a (RPL13a), and eukaryotic translation elongation factor 1 alpha 1 (eEF1A). 

### 2.9. In Vitro Study 

A transfection study using miRNA mimics was developed in order to verify the regulatory effect of miRNA on the in silico-predicted mRNA targets. Cardiac Muscle Cell Line (HL-1) (Sigma-Aldrich, St. Louis, MI, USA), an immortalized mouse cardiomyocyte cell line, were grown in 75 cm^2^ flasks using Claycomb medium (Sigma-Aldrich, St. Louis, MI, USA), supplemented with 100 μM norepinephrin, 10% fetal bovine serum (FBS) and 4 mM L-glutamine. HL-1 cells (2 × 10^5^) are seeded in 6-wells plates 24 h prior to transfection. 80 nM of synthetic miRNA mimics are transfected into cells using Lipofectamine 2000 (Table S3). After 6 h of transfection, the medium was replaced with Claycomb media and, 48 h later, the HL-1 cells were washed twice with PBS and used for total RNA extraction by *miRNeasy mini kit*. The reverse transcription was performed using 1 μg of total RNA obtained from HL-1 cells using *IScript cDNA Synthesis Kit* (Bio-Rad Laboratories, Hercules, CA, USA). The expression of miRNA putative target genes (cTns) was analyzed in HL-1 by CFX-96 Real-Time PCR detection systems (Bio-Rad), starting from 2 μL cDNA template and *SsoFAST™ EvaGreen^®^ supermix* (Bio-Rad Laboratories, Hercules, CA, USA). Primers for candidate reference genes and cardiac troponins were designed using Primer-Blast (National Center for Biotechnology Information) (Table S4). Normalization of cTns mRNA was carried out using hypoxanthine phosphoribosyltransferase 1 (HPRT1) as a reference gene. 

### 2.10. Statistical Analysis

Statistical analysis was performed using StatView 5.0.1 software (SAS Institute, Inc., Cary, NC, USA). Quantitative data were presented as median and interquartile range (I-III). Due to a non-normal distribution of circulating biomarkers, the original data were ln-transformed and parametric tests were used. Differences of cardiac miRNA expression and of their predicted targets (cTns gene) between patient groups were assessed by Mann-Whitney U test. Correlation among cardiac miRNAs and predicted targets expression was evaluated by Spearman correlation. Comparison between control and transfected cells was performed by paired t-student test. A *p*-value ≤ 0.05 was considered significant.

## 3. Results

### 3.1. Clinical Characteristics of HF Pediatric Patients

Imaging and laboratory characteristics of all 13 HF patients at baseline (pre-VAD) and at the time of VAD explant (heart transplant) were reported in Table 1. The median age was 29 months, six children were males, and the median weight was 9 kg. Overall, VAD therapy determined a significant improvement of cardiac function as assessed by echocardiography (Table 1). LVEF% (*p* = 0.0273) and right ventricle fractional area change (RVFAC%, *p* = 0.049) increased significantly at the time of VAD explant, together with a significant decrease of left ventricular end diastolic volume (LVEDV, *p* = 0.0087) and left ventricle end systolic volume (LVESV, *p* = 0.0201) (Table 1). At the time of heart transplant all the analyzed bio-humoral markers were unmodified by VAD support when compared to pre-VAD, except for total bilirubin plasma levels (*p* = 0.0161) (Table 1).

### 3.2. miRNA Profiling

**Figure 2:**
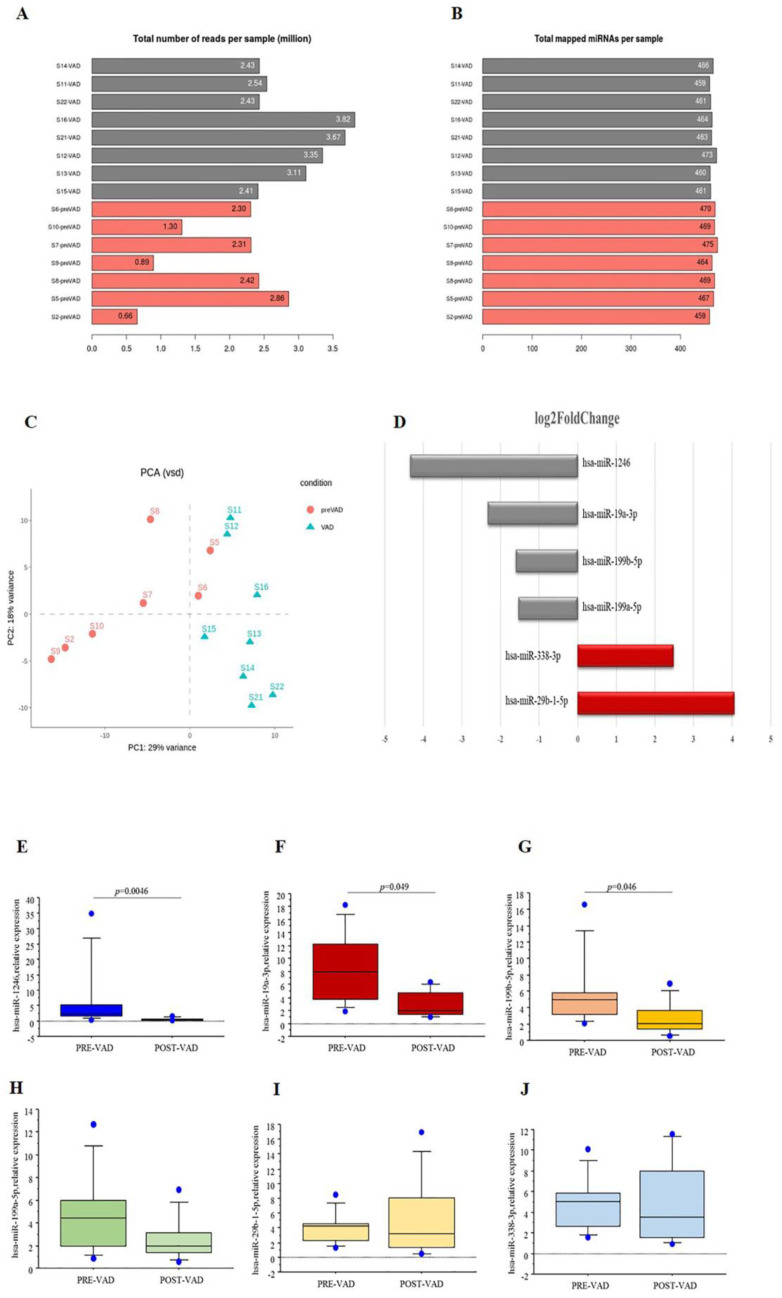
(**A**) Total number reads per sample. (**B**) Total mapped miRNAs per samples. (**C**) The distribution of miRNA among pre-VAD and post-VAD groups represented by Principal component analysis. (**D**) Differentially expressed cardiac miRNA from NGS experiments. Log_2_ Fold Changes between pre- and post-VAD are showed (grey bars: downregulated miRNA; red bars: up-regulated miRNAs). Real-time PCR data analysis of differentially expressed (**E**) hsa-miR-1246, (**F**) hsa-miR-19a-3p, (**G**) hsa-miR-199b-5p, (**H**) hsa-miR-199a-5p, (**I**) hsa-miR-29b-1-5p, (**J**) hsa-miR-338-3p in cardiac samples of HF children at pre-VAD compared to post-VAD.

NGS technology was employed to obtain miRNAs profile in cardiac samples collected from HF children before VAD implant and at the moment of heart transplant after VAD support. Overall, an average of 463 miRNAs were identified in each sample (Figure 2A,B). The distribution of the pre-VAD and post-VAD samples was showed in Figure 2C. A total of 6 cardiac miRNAs were differentially expressed in cardiac samples at pre-VAD compared to post-VAD (padj < 0.05). Among these, four miRNAs were downregulated, whilst two miRNAs were upregulated after VAD (Figure 2D). In the attempt to validate the NGS results, the cardiac expression levels of six selected miRNAs were evaluated by real-time PCR (Figure 2E–J). The expression of hsa-miR-1246, hsa-miR-19a-3p and hsa-miR-199b-5p decreased significantly after VAD, confirming the sequencing results. These three miRNAs were selected for downstream analysis. 

### 3.3. In-Silico Prediction of Putative miRNA Targets

**Figure 3:**
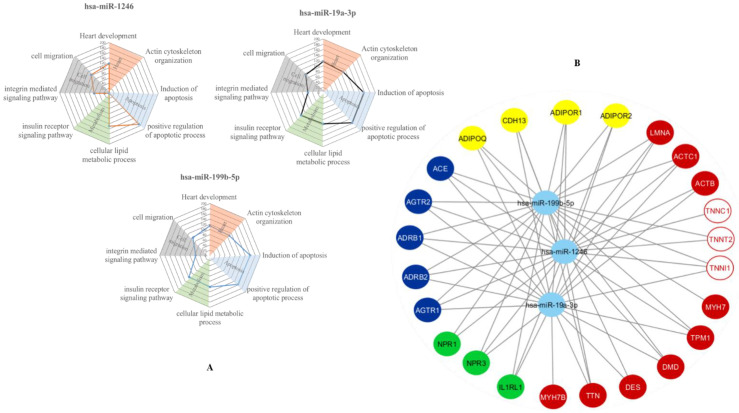
In-silico analysis. (**A**) Radar charts of the relevant biological processes simultaneously modulated by the selected cardiac miRNAs after gene ontology biological processes (GOBP) analysis. The levels show the proportion of the number of putative gene targets for each class of biological process. (**B**) Putative gene targets of selected miRNAs. The light blue circles represent the three validated sequencing miRNA, the yellow circles indicate the gene related to adiponectin system; the blue circle represent the genes involved in angiotensin and adrenergic systems; the green circle indicate natriuretic peptides receptors and Suppressor tumorigenicity 2; the red circles represent the sarcomere genes and the circle white and red the troponin genes. Arrows indicate the relationship between each miRNA and its putative targets.

An in-silico analysis was performed by miRWalk2 database to understand which are the potential biological pathway and putative gene targets for miRNAs validation. The analysis of biological functional patterns (Gene ontology terms) showed that at least two miRNAs are synergistically involved in a total of 52 processes, including heart development, actin cytoskeletal organization, apoptotic process regulation, metabolism regulation and cell migration (Figure 3A). Moreover, miRWalk2 database was used to identify the putative miRNA gene targets. Data obtained suggested that most of the putative target of validated miRNAs were implicated in cardiac sarcomere function (i.e., cardiac troponins, alpha/beta actin, myosin, etc.), cardiac protection (adiponectin system) and neurohormonal cardiomyocytes regulation (angiotensin system, natriuretic peptide receptors, adrenergic system) (Figure 3B). Among them, the focus of this study was the expression and regulation of cTns by VAD-modified miRNAs.

### 3.4. Cardiac Troponin Complex

**Figure 4:**
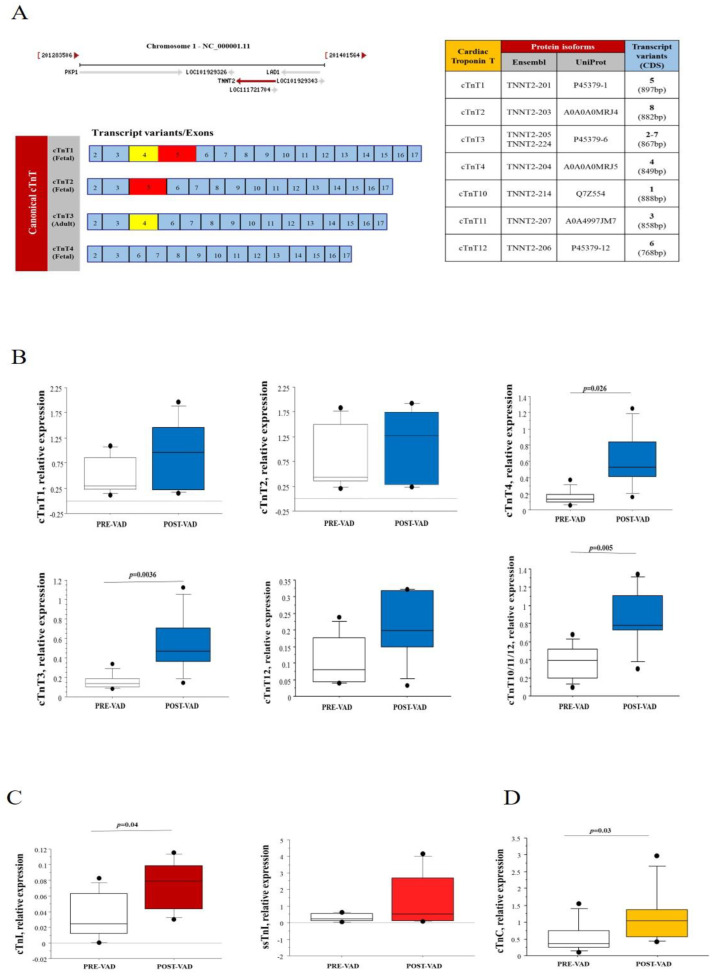
(**A**) Human cardiac troponin T gene (TNNT2) obtained from www.ncbi.nlm.nih.gov/gene, are at the top left, the canonical transcript variants of cardiac troponin T (cTnT) are at the lower left, fetal variants cTnT1, cTnT2 and cTnT4 and adult variant cTnT3 were obtained by splicing of exons 4 and/or 5. All canonical and non-canonical transcript variants and corresponding protein isoforms matched using Uniprot and Ensembl database in the table at the right. (**B**) Changes in canonical and non-canonical cTnT expression levels in HF children supported by VAD: comparison among pre-VAD and post-VAD samples. (**C**) Changes in cardiac troponin I (cTnI) and slow-skeletal troponin I expression levels in pediatric patients with HF treated with VAD: comparison among pre-VAD and post-VAD samples. (**D**) Comparison of cardiac troponin C expression levels between pre-VAD and post-VAD samples obtained from children with HF supported by VAD.

Uniprot and Ensembl analyses identified canonical (cTnT1, cTnT2, cTnT3, and cTnT4) and non-canonical (cTnT10, cTnT11, cTnT12) isoforms of cardiac Troponin T (Figure 4A). The mRNA levels of adult isoform of cTnT (cTnT3), fetal isoform cTnT4, and non-canonical isoforms cTnT 10/11/12 were significantly higher at post-VAD compared to pre-VAD, while no differences were observed for the other fetal isoforms cTnT expression (Figure 4B). Both adult cardiac troponin I (cTnI; TNNI3 gene) and fetal/slow skeletal troponin I (ssTnI; TNNI1 gene) were expressed in the cardiac tissue of HF children, but only cTnI expression levels increased significantly by mechanical support compared to pre-VAD levels (Figure 4C). The mRNA levels cardiac of troponin C (cTnC) (that consist in only one isoform during all life phase in human) increased after the VAD similarly to some other components of troponin complex (Figure 4D). 

The expression levels of troponin complex were evaluated also in a group of HF adult patients at pre- and post-VAD. Clinical features of adult group were reported in Table S5. mRNA levels of all variants of cTnT significantly increased after VAD in adult patients, together with cTnI and cTnC, while ssTnI mRNA was no longer expressed after VAD support in HF adult patients (Table 2). When the mRNA levels of troponin complex were compared between pediatric and adult patients, gene expression of cTnI, cTnT1, cTnT3, cTnT4, cTnT 10/11/12 and cTnC were significantly higher in HF adults compared to HF children, both pre-VAD and post-VAD (Table 2). No differences were found in ssTnI expression among adult and children at pre-VAD, whilst post-VAD ssTnI was still expressed in children but not in adults (Table 2). 

### 3.5. Comparison of Expression Levels of Validate Cardiac miRNAs and Cardiac Troponin Complex in HF Children Supported by VAD

The potential relationship among validated miRNA and some sarcomere proteins in pediatric patients with HF supported by VAD was evaluated. Cardiac expression levels of hsa-miR-1246, hsa-miR-19a-3p and hsa-miR-199b-5p were negatively related to cTnI, cTnT3, cTnT4, whilst only hsa-miR-1246 and hsa-miR-19a-3p were negatively related to cTnC (Table 3). 

### 3.6. In Vitro Validation of Putative Targets of Selected Cardiac miRNAs

**Figure 5:**
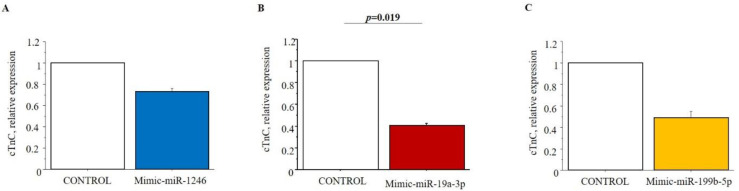
In vitro study results. Cardiac troponin C (cTnC) expression levels in HL-1 cell line transfected with (**A**) mimic-miR-1246; (**B**) mimic-19a-3p; (**C**) mimic-199b-5p compared to control (no treated cell). Comparison between control and transfected cells was performed by paired Student’s t-test.

HL-1 cells were used to test the possible regulatory role of selected miRNAs on cTnI, cTnT and cTnC by miRNA-mimic transfection study. Only the expression levels of cTnC were modified in cells 48 h after transfection with miRNA mimic. Specifically, the cTnC levels in HL-1cells transfected with hsa-miR-19a-3p mimic significantly decreased compared to negative control (Figure 5).

## 4. Discussion 

Understanding the molecular pathways involved in HF pathophysiology in children and unveiling the effects of VAD implant could be of great importance to identify new therapeutic strategies to reduce disease progression in pediatric patients. Accordingly, changes in cardiac miRNA profile in HF children supported by VAD were described for the first time in this pilot study. Moreover, the effects of selected miRNAs on putative target were investigated. Specifically, we found that:

- Cardiac miRNA profile was modified in HF children by VAD support, as assessed by NGS and real-time PCR;
- The re-expression of fetal isoforms of cTnT and ssTnI can be observed in cardiac tissue from HF children;
- The NGS-identified miRNAs were involved in the regulation of troponin complex, as confirmed by the in vitro study.

Growing evidence has shown that miRNAs are extensively involved in the pathogenic mechanisms that lead to HF in adult patients, such as hypertrophy, apoptosis and fibrosis [20,21]. 

Matkovich et al. identified 28 upregulated miRNAs in the end-stage congestive failing myocardium of adults and suggested their potential association with HF pathophysiology [22]. Levels of 250 miRNAs were modified in HF adult patients and their expression were etiology-related (DCM or HCM) [23]. However, from a molecular point of view, HF in the child must be considered as a completely separate condition with respect to the adult [24]. For this reason, the data collected on HF adult cannot be used for pediatric patients. Data of miRNAs in HF pediatric patients are extremely scarce. Stauffer et al. found that 17 miRNAs were significantly regulated in pediatric failing heart compared to healthy children and that the majority of these miRNAs are not modified or negatively regulated in hearts of HF adult patients [14]. In this work, for the first time, the profiling of miRNAs from cardiac samples of HF children before and after VAD support was evaluated. After sequencing and validation phases, the three miRNAs, hsa-miR-199b-5p, hsa-miR-19a-3p and hsa-miR-1246, were found differentially expressed in HF children treated with VAD. Data from literature suggest a role in cardiac hypertrophy regulation for miR-199b-5p by activation of activator of calcineurin/nuclear factor of activated T-cell (can/NFAT) signaling [24,25]. Moreover, the use of inhibitors of miR-199b-5p reduced ventricular remodeling and preserved the cardiac contractility in animal model of pressure overload [24]. In adult HF patients, low cardiac levels of hsa-miR-19a-3p/19b-3p were associated with autophagy activation [26]. In addition, in vivo studies suggest that decreased miR-19a-3p expression could be associated with increased of cardiac hypertrophy by phosphodiesterase 5A (PDE5A) and myocyte enhancer factor 2 (MEF2) regulation [27,28]. No data are present in the literature that describing the role of miR-1246 in heart remodeling. Overall, until now, no works described the implication of hsa-miR-199b-5p, hsa-miR-19a-3p and hsa-miR-1246 in molecular mechanisms underlying HF in pediatric patients. In this study, the use of in-silico analysis suggested that hsa-miR-199b-5p, hsa-miR-19a-3p and hsa-miR-1246 could be potentially involved in the regulation of sarcomere elements expression and consequently could interfere with the heart’s ability to contract. The predicted association among the validated miRNAs and sarcomere genes are in agreement with the DCM etiology of HF for most of the enrolled patients, considering that altered gene expression of sarcomere proteins is the essential condition of DCM [29].

Among sarcomere proteins, more specifically in this work, the cardiac troponin complex in HF children was studied evaluating the mRNA expression levels and their regulation by the NGS-identified miRNAs before and after VAD support. It is well known that during the human heart development, different isoforms of cTnT and variants of TnI are physiologically expressed and these molecules are involved in regulation of sarcomere organization [30]. In vitro studies found that the co-existence of multiple cTnT and TnI isoforms can causes a desynchronized sarcomere function and impairs cardiac efficiency, proportionally to degree of cTnT-TnI heterogeneity [30]. Data from literature show that in adult HF patients many fetal variants of cardiac troponin are re-expressed [31] but no data on pediatric patients are present. We observed that all fetal and adult cTnT and TnI isoforms were expressed in pediatric and adult patients with HF, possibly confirming that the co-existence of these isoforms reduces the ability of the heart to properly work, in agreement with data previously reported [30]. Moreover, after VAD treatment, a significant increase of only cTnT3, cTnT4, cTnI and cTnC expression levels was observed in children, suggesting a possible effect of VAD support on sarcomere organization in children but not in adult patients. This different response to treatment between two groups may be dependent on the age of patients, on a different etiology of the disease between the two groups or on different epigenetic regulation of cardiac troponin genes expression. Finally, by evaluating the cardiac miRNA levels together with the expression levels of cardiac troponin complex in HF children, a negative correlation among of hsa-miR-1246, hsa-miR-19a-3p and hsa-miR-199b-5p and cTnI, cTnT3, cTnT4 and cTnC was found. Furthermore, in vitro studies on HL-1 cells confirmed a regulatory role only for hsa-miR-19a-3p on cTnC. cTnC plays a critical role in heart and skeletal muscle. During muscle activation, calcium binding to cTnC favors the binding to troponin I, removing adjacent inhibitory regions of troponin I from actin and allowing muscle contraction to proceed [32]. In HF children supported by VAD, an increased in cTnC mRNA expression and a reduction of hsa-miR-19a-3p could ensure a better cardiac contraction.

This study has some limitations. Due to the lack of cardiac tissue from healthy pediatric subjects, it was not possible to perform a comparison between miRNAs and relative target expression of healthy children and HF pediatric patients. Moreover, the number of HF pediatric patients is limited, but the internal control operated by collecting in the same patient myocardial tissue from both LV and septum allowed a better interpretation of the results. 

In summary, results from this study could suggest that, in children, the effect of VAD on sarcomere organization may be managed through miRNA expression, adding a new layer of regulation to molecular mechanisms of heart remodeling. Future investigations performed in a large population are necessary to confirm the regulatory role in cardiac contraction of hsa-miR-1246, hsa-miR-19a-3p and hsa-miR-199b-5p and their potential use as therapeutic targets in HF children.

## Figures and Tables

**Table 1:** Clinical features of HF children at the moment of VAD implant (Pre-VAD) and the moment of heart transplant (Post-VAD).

	HF Children Pre-VAD	HF Children Post-VAD	*p*-Value
*Age*, *months*	29 (5–123)	-	
*Male gender (n)*	6 (13)	-	
*Etiology*, *n (%)*			
*DCM*	69.2%	-	
*LV non-compaction*	15.4%	-	
*RCM*	15.4%	-	
*Weight*, *Kg*	9 (4.8–26)	-	
*LVEF*, *%*	19 (13.75–20.75)	37 (26–45)	*p* = 0.0273
*LVEDV*, *mL*	57 (37.5–83)	24 (15.25–30.75)	*p* = 0.0087
*LVESV*, *mL*	37 (28.7–71)	14 (8.72–20)	*p* = 0.0201
*LVEDD*, *mm*	47 (42.5–56.5)	41 (30.5–49.5)	ns
*LVESD*, *mm*	44.5 (39–57)	37 (23–45)	ns
*TAPSE*, *mm*	0.985 (0.7–1.25)	0.72 (0.63–1.015)	ns
*RVFAC*, *%*	33 (23.5–41)	45 (40.5–56.25)	*p* = 0.049
*White blood cells*, *× 10^9^/L*	9.2 (7.76–12.7)	8.77 (8.07–9.65)	ns
*Hb*, *g/dL*	12.9 (12.4–14.2)	11.7 (10.3–12.67)	ns
*Platelets*, *× 10^9^/L*	272.5 (204–289)	327 (252–403)	ns
*INR*	1.2 (1.14–1.2)	1.1 (1–2.55)	ns
*Urea nitrogen*, *mg/dL*	15.5 (12.7–32.5)	21 (10–31.25)	ns
*Creatinine*, *mg/dL*	0.43 (0.275–0.615)	0.37 (0.2–0.5)	ns
*Albumin*, *g/dL*	4.2 (3.6–5.25)	4.1 (3.9–4.4)	ns
*Glucose*, *mg/dL*	113 (71–128)	91 (87.75–120.75)	ns
*C-reactive Protein, mg/dL*	0.41 (0.11–1.5)	0.8 (0.2–1.7)	ns
*Bilirubin tot*, *mg/dL*	1.2 (0.51–1.95)	0.36 (0.25–0.72)	*p* = 0.0161
*Lactate Dehydrogenase*, *U/L*	812 (405–1011)	742 (634–1088)	ns

DCM: dilated cardiomyopathy; LV non-compaction: left ventricular non-compaction; RCM: restrictive cardiomyopathy. LVEF: left ventricular ejection fraction; LVEDV: left ventricular end diastolic volume; LVESV: left ventricular end systolic volume; LVEDD: left ventricular end diastolic diameter; LVESD: left ventricular end systolic diameter; TAPSE: tricuspid annular plane systolic excursion; RVFAC: right ventricular fractional area change; Hb: hemoglobin; INR: International Normalized Ratio.

**Table 2:** Comparison of cardiac troponin complex expression levels among HF children and HF adult patients before (Pre-VAD) and after (Post-VAD) VAD support.

	Pre-VAD			Post-VAD		
	Children	Adult	*p*-Value	Children	Adult	*p*-Value
*cTnI*	0.03 ± 0.009	0.38 ± 0.08	*p* < 0.0001	0.07 ± 0.012	5.06 ± 1.75 *	*p* = 0.0045
*ssTnI*	0.3 ± 0.096	0.58 ± 0.38	ns	0.77 ± 0.49	---	---
*cTnC*	0.58 ± 0.176	8.87 ± 1.57	*p* < 0.0001	1.179 ± 0.328	16.79 ± 3.8 *	*p* = 0.0045
*cTnT 1*	0.48 ± 0.13	1 ± 0.13	*p* = 0.01	0.89 ± 0.27	4.27 ± 1.17 *	*p* = 0.006
*cTnT 2*	0.86 ± 0.22	1.08 ± 0.18	ns	1.04 ± 0.29	8.06 ± 2.79 *	*p* = 0.008
*cTnT 3*	0.16 ± 0.03	0.88 ± 0.9	*p* < 0.0001	0.54 ± 0.12	2.8 ± 0.6 *	*p* = 0.0074
*cTnT 4*	0.15 ± 0.032	0.866 ± 0.09	*p* < 0.0001	0.6 ± 0.14	1.66 ± 0.24 *	*p* = 0.007
*cTnT 12*	0.11 ± 0.03	0.79 ± 0.09	*p* < 0.0001	0.22 ± 0.04	0.82 ± 0.27	*p* = 0.0045
*cTnT 10*,*11*,*12*	0.37 ± 0.066	1 ± 0.129	*p* = 0.0002	0.84 ± 0.13	5.08 ± 1.84 *	*p* = 0.0017

* *p* < 0.001 comparison between pre-VAD vs. post-VAD in adult group. cTnI: cardiac troponin I; ssTnI: slow skeletal troponin I; cTnC: cardiac troponin C; cTnT 1: cardiac troponin T isoform 1; cTnT 2: cardiac troponin T isoform 2, cTnT 3: cardiac troponin T isoform 3; cTnT 4: cardiac troponin T isoform 4; cTnT 12: cardiac troponin T isoform 12; cTnT 10,11,12: cardiac troponin T isoforms 10,11,12.

**Table 3:** Relation among cardiac miRNA and cardiac troponin expression levels in HF children supported by VAD.

	hsa-miR-1246	hsa-miR-19a-3p	hsa-miR-199b-5p
*cTnI*	Rho = −0.744 *p* = 0.004	Rho = −0.853 *p* = 0.001	Rho = −0.591 *p* = 0.022
*ssTnI*	ns	Rho = −0.808 *p* = 0.0051	ns
*cTnC*	Rho = −0.686 *p* = 0.01	Rho = −0.625 *p* = 0.0194	ns
*cTnT1*	ns	ns	ns
*cTnT2*	ns	ns	ns
*cTnT3*	Rho = −0.771 *p* = 0.0028	Rho = −0.706 *p* = 0.0063	Rho = −0.562 *p* = 0.0296
*cTnT4*	Rho = −0.791 *p* = 0.0022	Rho = −0.732 *p* = 0.0046	Rho = −0.624 *p* = 0.0157

## Data Availability

The data that support the findings of this study are available from OPBG and IFC-CNR but restrictions apply to the availability of these data, which were under authorization for the current study and in compliance with GDPR 2016/679, and so are not publicly available. Data are however available from the corresponding author on reasonable request and with permission of OPBG and IFC-CNR.

## Supplementary Materials

The following are available online at https://www.mdpi.com/article/10.3390/biomedicines9101409/s1, Table S1: Primer sequences for miRNAs, Table S2: Primer sequences for human cardiac troponin complex and reference genes, Table S3: miRNA mimics for HL-1 cell line transfection study, Table S4: Primer sequences for HL-1 cell line cardiac troponin complex and reference genes, Table S5: Clinical features of HF adult patients at the moment of VAD implant (Pre-VAD).
