# Supplementary material for: Epigenetic Regulation of Cardiac Troponin Genes in Pediatric Patients with Heart Failure Supported by Ventricular Assist Device
Source: Biomedicines. 2021 Oct 7;9(10):1409. doi: 10.3390/biomedicines9101409 (PMC8533380; doi:10.3390/biomedicines9101409)
Supplement: Supplementary file 1 [file biomedicines-09-01409-s001.zip › biomedicines-1399296-supplementary.pdf]

Supplementary material

Supplementary Table S1: Primer sequences for miRNAs

miRNAs	Sequence	MIMAT NUMBER
**hsa-miR-1246**	AATGGATTTTTGGAGCAGG	MIMAT0005898
**hsa-miR-19a-3p**	TGTGCAAATCTATGCAAAACTGA	MIMAT0000073
**hsa-miR-199b-5p**	CCCAGTGTTTAGACTATCTGTTC	MIMAT0000263
**hsa-miR-199a-5p**	CCCAGTGTTCAGACTACCTGTTC	MIMAT0000231
**hsa-miR-338-3p**	TCCAGCATCAGTGATTTTGTTG	MIMAT0000763
**hsa-miR-29b-1-5p**	GCTGGTTTCATATGGTGGTTTAGA	MIMAT0004514
**U6**	CGCAAGGATGACACGCAAATTC	-----

Supplementary Table S2: Primer sequences for human cardiac troponin complex and reference genes

Gene	Sequence		N. GenBank access
**cTnI**	Forward	CTGCGGAGAGTGAGGATCTC	NM_000363.4
	Reverse	GTCCTCCTTCTTCACCTGCT	
**ssTnI**	Forward	ATGCCGGAAGTCGAGAGAAAA	NM_003281.3
	Reverse	TCGTATCGCTCCTCATCCAC	
**cTnC**	Forward	CATCTACAAGGCTGCGGTAG	NM_003280.3
	Reverse	ACGAAGATGTCGAAGGCTGC	
**cTnT 1**	Forward	AGGACTGGAGAGAGGACGAAGACGA	NM_000364.3<br>NM_001276346.1<br>NM_001276345.1
	Reverse	GGACTCCTCCATTGGGCCAT	
**cTnT 2**	Forward	GAGGAGCAGGAAGAAGAGGAGGAC	A0A0A0MRJ4
	Reverse	GGTCTCAGCCTCTGCTTCAGCATC	
**cTnT 3**	Forward	AGAAGCAGCTGTTGAAGAGCAGG	NM_001276347.1<br>NM_001001430.2
	Reverse	GGACTCCTCCATTGGGCCAT	
**cTnT 4**	Forward	GAGGAGGAGGAGCAGGAAGAGCA	NM_001001432.2
	Reverse	GGACTCCTCCATTGGGCCAT	
**cTnT 12**	Forward	TGGAGAGAGAGTGGACTTTGATGAGAGA	NM_001276346.1
	Reverse	CATCCTCAGCCTTCCTCCTGTTCT	
**cTnT 10,11,12**	Forward	GGGGGTTACATCCAGAAGACAGAG	NM_001276346.1<br>NM_000364.3<br>NM_001001431.2
	Reverse	TCAGGTGGTCAATGGCCAGCAC	
**eEF1A**	Forward	CTTTGGGTCGCTTTGCTGTT	NM_001402
	Reverse	CCGTTCTTCCACCACTGATT	
**RPL13a**	Forward	CGCCCTACGACAAGAAAAAG	NM_012423
	Reverse	CCGTAGCCTCATGAGCTGTT	
**YWHAZ**	Forward	ATGCAACCAACACATCCTATC	NM_00113572
	Reverse	GCATTATTAGCGTGCTGTCTT	

Supplementary Table S3: miRNA mimics for HL-1 cell line transfection study

miRNAs	mimic
miR-1246	5′-AAUGGAUUUUUGGAGCAGGUU-3′<br>3′-UUUCCCC UAAAAA CCUCGUCC-5′
miR-19a-3p	5′-UGUGCAAAUCUAUGCAAAACUGAUU-3′<br>3′-UUAAUCGUUUAGAUACGUUUUGACU-5′
miR-199b-5p	5′-CCCAGUGUUUAGACUACCUGUUCUU-3′<br>3′-UUGAAUCACAAAUCUGAUGGACAAG-5′
miR-CT	5′-CUCUAGGUUAAACUCCUGGUU-3′<br>3′-UUGUAAUCCAAUUUGAGGACCAA-5′

Supplementary Table S4: Primer sequences for HL-1 cell line cardiac troponin complex and reference genes

**Gene**	**Sequence**		**N. GenBank access**
**cTnI**	Forward	GATGCGGCTGGGGAACC	NM_009406.4
	Reverse	ACTTTTTCTTGGCGTGTGGC	
**ssTnI**	Forward	TGCTTTGGTCCCTTTCCAGCC	NM_001112702.1
	Reverse	CCCCTTTGTGCGCCATTTCAT	
**cTnC**	Forward	AGCTGCGGTAGAACAGTTGA	NM_009393.3
	Reverse	CCACTGCCATCCTCGTCTAC	
**cTnT**	Forward	TTCGACCTGCAGGAAAAGTT	NM_001130174.2
	Reverse	CTTCCCACGAGTTTTGGAGA	
**HPRT**	Forward	GGAGGGGTAGCACCTCCT	NM_013556.2
	Reverse	AACCTGGTTCATCATCGCTAA	

Supplementary Table S5: Clinical features of HF adult patients at the moment of VAD implant (Pre-VAD)

	**HF adults**<br><br>**Pre-VAD**
**Age, years**	60 (50-64)
**Male gender (n)**	14(16)
**Weight, (Kg)**	69 (65.5-80.5)
**LVEF,(%)**	22.5 (19.5-25)
**LVEDV,(mL)**	235 (181.25-291.25)
**LVESV,(mL)**	176.5 (140-229)
**LVEDD,(mm)**	67 (63.25-71.75)
**TAPSE,(mm)**	15 (14-17.125)
**White blood cells**	8.55 (6.15-12.75)
**Hb**	12.25 (10.65-13.5)
**Platelets**	228.5 (152.5-287)
**Urea nitrogen, mg/dL**	56 (43-66)
**Creatinine, mg/dL**	1.195 (0.920-1.525)
**C-reactive Protein, mg/dL**	1.4 (0.45-6.95)
**Bilirubin tot, mg/dL**	1.17 (0.66-1.69)
**INR**	1.2 (1.1-1.4)
